# Wei2GO: weighted sequence similarity-based protein function prediction

**DOI:** 10.7717/peerj.12931

**Published:** 2022-02-15

**Authors:** Maarten J.M.F. Reijnders

**Affiliations:** 1 Department of Ecology and Evolution, University of Lausanne, Lausanne, Switzerland; 2 Swiss Institute of Bioinformatics, Lausanne, Switzerland

**Keywords:** Gene ontology, Protein functions, Sequence similarity, Open source, Proteins

## Abstract

**Background:**

Protein function prediction is an important part of bioinformatics and genomics studies. There are many different predictors available, however most of these are in the form of web-servers instead of open-source locally installable versions. Such local versions are necessary to perform large scale genomics studies due to the presence of limitations imposed by web servers such as queues, prediction speed, and updatability of databases.

**Methods:**

This paper describes Wei2GO: a weighted sequence similarity and python-based open-source protein function prediction software. It uses DIAMOND and HMMScan sequence alignment searches against the UniProtKB and Pfam databases respectively, transfers Gene Ontology terms from the reference protein to the query protein, and uses a weighing algorithm to calculate a score for the Gene Ontology annotations.

**Results:**

Wei2GO is compared against the Argot2 and Argot2.5 web servers, which use a similar concept, and DeepGOPlus which acts as a reference. Wei2GO shows an increase in performance according to precision and recall curves, F_max_ scores, and S_min_ scores for biological process and molecular function ontologies. Computational time compared to Argot2 and Argot2.5 is decreased from several hours to several minutes.

**Availability:**

Wei2GO is written in Python 3, and can be found at https://gitlab.com/mreijnders/Wei2GO.

## Introduction

Predicting proteins functions computationally is an integral part of omics research, due to the laborious process of experimentally determining protein functions. This is most apparent in the size difference of Uniprot’s TrEMBL and SwissProt databases, which contain predicted and manually curated proteins respectively (The UniProt Consortium, 2019). Because of the dependence on predicted protein functions, many software tools have been developed in an attempt to improve the accuracy of electronically annotating these proteins with Gene Ontology (GO) terms (Ashburner et al., 2000; The Gene Ontology Consortium, 2019).

A comprehensive overview of protein function prediction methods is the Critical Assessment of Functional Annotation (CAFA) competition (Zhou et al., 2019). In its most recent edition, CAFA3, it compared the predictions of 68 participants employing a wide variety of techniques including sequence homology, machine learning, and text mining amongst others. The top-performing predictors in this challenge are considered the state-of-the-art for protein function prediction. With this exposure and proven performance, predictors participating in CAFA have the potential to act as a resource for any user wanting to predict protein functions of their organism of interest. But for CAFA participants to be such a resource, they have two other factors to consider: accessibility and speed. A look at the top performers of CAFA shows that nine out of fourteen teams which placed top ten in one of the GO ontologies, provide their method as a tool accessible through a web server. However, only four out of fourteen teams provide a working locally installable option. Web servers provide an excellent option for the annotation of a limited amount of proteins, but larger amounts are problematic due to computational limitations.

Contrary to web servers, locally installable software provides opportunities to speed up predictions without limitations usually imposed, such as limited protein sequence inputs or queues shared with multiple users. Local versions provide users the means to update the software databases, which is a necessity to provide the most up-to-date and accurate predictions. As a bonus, these versions are often open-source and provide flexibility in the usage of the tool by advanced users, *e.g*. by utilizing a meta-predictor (Reijnders & Waterhouse, 2019). As such, there is a need for accurate open-source and locally installable prediction tools.

This paper describes Wei2GO: a weighted sequence similarity and python-based open-source function prediction software. Wei2GO utilizes DIAMOND (Buchfink, Xie & Huson, 2015) and HMMER (HMMER, 2021) searchers against the UniProtKB (The UniProt Consortium, 2019) and Pfam (El-Gebali et al., 2019) databases respectively, acquires GO terms through these homology searches, and calculates several weighted scores to accurately associate probabilities to these GO terms. Wei2GO is similar in concept to the web server-based Argot2.5 (Lavezzo et al., 2016, p. 2) and its predecessor Argot2 (Falda et al., 2012, p. 2), which were top performers in CAFA3 and CAFA2 respectively (Radivojac et al., 2013; Zhou et al., 2019). Due to this shared niche, this paper provides comparisons between these tools and Wei2GO using precision recall curves, F_max_ scores, S_min_ scores, and a comparison of computational time for their predictions.

## Methods

Wei2GO is a function prediction tool based on sequence similarity with reference proteins, using adapted methods from Argot2 and Argot2.5 to provide an open-source alternative and to improve predictions where possible. It uses two of the most common sequence alignment algorithms, DIAMOND and HMMER, against the UniProtKB and Pfam database respectively. Each sequence alignment hit is associated with GO terms, taken from the Gene Ontology Annotation (GOA) database (Huntley et al., 2015) for UniProtKB, and pfam2go (Blum et al., 2021) for Pfam. All GO terms associated with a hit are given a weight relative to their e-value and associated annotation evidence. The weights for each protein-GO term pair are summed across the DIAMOND and HMMER matches. Based on their semantic similarity, the weights of similar GO terms are obtained to get a group score. Finally the GO weight, its information content, and group score are used to calculate the final score associated with the prediction.

Equations outlined below are in agreement with those of Argot2 and Argot2.5 unless otherwise specified. The main algorithmic differences between Argot2, Argot2.5, and Wei2GO are:

1. The omission of a taxonomy filter used by Argot2.5.
2. The omission of supplementing Pfam sequence similarity hits with GO terms of non-Pfam origin.
3. The addition of a weight multiplier for GO terms derived from sequence similarity with UniProtKB proteins if these original terms are annotated with an evidence code other than IEA (Inferred from Electronical Annotation).

### Wei2GO input preparation

Wei2GO requires two input files:

1. Diamond or BLASTP hits against the UniProtKB database in tabular format (--outfmt 6).
2. HMMScan hits against the Pfam-A database as a table output (--tblout).

These files can either be produced separately, or be incorporated with Wei2GO *via* its Snakemake pipeline (Mölder et al., 2021).

### Wei2GO algorithm

Each protein matched with a UniProtKB reference *via* BLAST or DIAMOND is annotated with GO terms associated to these reference proteins in the GOA database. Consecutively, each protein matched with a Pfam domain *via* HMMScan is annotated with GO terms associated to this domain in the pfam2go database.

For every protein *v*, a GO term *i* is given a weight *W*(*v,i*) by taking the sum of the -log10 e-value *E* of all UniProtKB and Pfam sequence similarity hits *j* associated with this GO term, where e-values above 1 are filtered out:

(1) $$W\left( {v,i} \right) = \; \mathop \sum \limits_{j \in Hits\left( {v,i} \right)} - log10\left( {{E_j}} \right),\; \;$$

where *Hits*(*v,i*) is the set of BLAST and HMMScan sequence aligment hits to *v* annotated with GO term *i*, and ${E_j}$ is the e-value of the sequence alignment *j*. Unique to Wei2GO compared to Argot is that terms annotated to a UniprotKB protein with an evidence code other than IEA (Inferred from Electronical Annotation) are given a weight multiplier:

(2) $$W\left( {v,i} \right) = \; \mathop \sum \limits_{j \in Hits\left( {v,i} \right)} - log10\left( {{E_j}} \right)\cdot log2\left( P \right),$$

where *P* is the ratio between the number of GO terms annotated with an IEA *vs* GO terms annotated with an other evidence code in the GOA database. Given that the amount of GO terms annotated with an IEA code is substantially higher than those with non-IEA codes, *P* is larger than 1 and results in higher weights when based on non-IEA instead of IEA evidence codes.

In accordance with the GO Directed Acyclic Graph (DAG) structure all weights associated to child terms of a GO term are added to its weight, with the rationale that if a protein is annotated with a certain GO term it by default includes all its parent terms. This has the effect of assigning more confidence in GO terms higher up the DAG, *i.e*. annotations that are more generic and less prone to errors.

The total weight associated with each protein-GO term pair is used to calculate the Internal Confidence (InC) score by taking the relative weight of the GO term compared to the weight of the root node (3):

(3) $${\rm InC}\left( {{\rm v},{\rm i}} \right) = \; \displaystyle{{{\rm W}\left( {v,i} \right)} \over {W\left( {root} \right)}}.$$

The InC score is used to calculate a Group Score (GS) for each GO term *i* associated to a protein, by taking the sum of the InC of all GO terms *j* annotated to a protein *v*, if GO term *j* has a semantic similarity of 0.7 or higher with GO term *i*:

(4) $$GS\left( {v,i} \right) = \mathop \sum \nolimits_{j\ \varepsilon\ annotations\left( v \right)} \; InC\left( {v,j} \right)\cdot I\; \left( {Sim\left( {i,j} \right) \ge 0.7} \right),$$

where *I*(.) is the indicator function which is equal to 1 if its argument is true and 0 otherwise, and annotations(v) is the set of GO annotations for protein v. Note that each GO term can have a unique GS score, as opposed to Argot where one singular GS score is defined for a group of similar GO terms. The value of Sim (semantic similarity) is calculated using Lin’s formula (Lin, 1998) as:

(5) $$Sim\left( {i,j} \right) = \displaystyle{{2{\rm \; }\cdot {\rm \; IC}\left( k \right)} \over {IC\left( i \right) + IC\left( j \right)}},$$

where *k* is the first joint parent term between GO term *i* and *j*, and the IC (Information Content) is calculated as:

(6) $$IC\left( i \right) = \; - {\rm log}10(P\left( i \right),$$

where *P*(*i*) is the fraction of proteins annotated to term *i*. Finally, a total score (TS) for each GO term annotated to a protein *v* is calculated as:

(7) $$TS\left( {v,i} \right) = IC\left( i \right)\cdot \displaystyle{{{\rm InC}\left( {v,i} \right)} \over {GS\left( {v,i} \right)}}\cdot {\rm W}\left( {v,i} \right).\;$$

The TS score is similar to that of Argot2 and Argot2.5, which combines the scores calculated by the equations above. However, Argot introduces an additional non-cumulative InC score, *i.e*. Eq. (3) without adding the weights of child terms, to remove bias of scores towards terms which have a higher probability of being predicted, *i.e*. a lower IC. This is done in an attempt to reduce bias towards predicting GO terms that are generic at the cost of GO terms that are highly specific, as reflected by their IC. Wei2GO instead operates under the assumption that GO terms which are easier to predict due to any reason, including having a lower IC, should have this reflected in their score. Any possible negative effects of this bias towards terms with lower IC affecting terms with higher IC will be reflected in the ‘*S*_*min*_’ evaluation metric, which measures performance proportional to these IC’s (See ‘Test set evaluation’ section).

### Test set generation

To assess the performance of Wei2GO it is compared to Argot2.5 and its predecessor Argot2, with the addition of DeepGOPlus (Kulmanov & Hoehndorf, 2020) which acts as a reference point. All proteins in the GOA database created between 01-01-2018 and 01-06-2020 were extracted. Only GO terms with the evidence code EXP (Inferred from Experiment), IDA (Inferred from Direct Assay), IPI (Inferred from Physical Interaction), IMP (Inferred from Mutant Phenotype), IGI (Inferred from Genetic Interaction), IEP (Inferred from Expression Pattern), TAS (Traceable Author Statement), and IC (Inferred by Curator), were retained. All molecular function terms ‘GO:0005515’ (protein binding) were removed from the annotation set if it was the only molecular function term annotated to the protein, to remove a large amount of bias due to many proteins having this generic term as their only molecular function term. This same approach was used in the CAFA3 test set generation.

The test set was expanded by including all terms according to their GO DAG structure using the ‘is_a’ and ‘part_of’ relationships for parent terms. The final test set consists of 4,067 proteins with 97,054 assigned GO terms of which 1,922 proteins have 59,697 biological process terms, 651 proteins have 4,785 molecular function terms, and 2,387 proteins have 32,572 cellular component terms.

### Test set evaluation

Precision and recall is used for the assessment of Wei2GO. For a threshold *t*, the precision *pr* and recall *rc* are calculated per protein *v*, ${T_v}$ is the set of true GO terms for a protein *v*, ${P_v}$ is the set of predicted GO terms for a protein *v*, *m(t)* is the number of proteins with at least one GO term predicted with a score above the threshold, and *n* is the total number of proteins:

(8) $$p{r_v}\left( t \right) = \displaystyle{{\left| {{P_v}\; \cap \; {T_v}} \right|} \over {\left| {{P_v}} \right|}},$$

(9) $$r{c_v}\left( t \right) = \; \displaystyle{{\left| {{P_v}\; \cap \; {T_v}} \right|} \over {\left| {{T_v}} \right|}},$$

(10) $$avgPr\left( t \right) = \; \displaystyle{1 \over {m\left( t \right)}}\; \cdot \; \mathop \sum \limits_{v = 1}^{m\left( t \right)} p{r_v}\left( t \right),$$

(11) $$avgRc\left( t \right)\; = \; \displaystyle{1 \over n}\; \cdot \; \mathop \sum \limits_{v = 1}^n r{c_v}\left( t \right).$$

The optimal balance between precision and recall is calculated using the maximum F1-score, which for this study is calculated by calculating F1-scores with 0.01 step-sizes after transforming the Total Scores to a 0–1 range:

(12) $${F_{max}} = \mathop {\max }\limits_t \left\{ {\displaystyle{{2\cdot avgPr\left( t \right)\cdot avgRc\left( t \right)} \over {avgPr\left( t \right) + avgRc\left( t \right)}}} \right\}.$$

The *S*_*min*_ score is calculated based on the remaining uncertainty *ru* and missing information *mi*, which is the IC of all false negatives and false positives, respectively. A threshold *t* step-size of 0.01 is used after transforming the Total Scores to a 0–1 range.

(13) $${S_{min}} = \mathop {\min }\limits_t \sqrt {ru{{\left( t \right)}^2} + mi{{\left( t \right)}^2},}$$

with *ru* as:

(14) $$ru\left( t \right) = \displaystyle{1 \over n}\mathop \sum \limits_{v = 1}^n \mathop \sum \nolimits_{GO \in {T_v} - {P_v}\left( t \right)} IC\left( i \right),$$

and *mi* as:

(15) $$mi\left( t \right) = \displaystyle{1 \over n}\mathop \sum \limits_{v = 1}^n \mathop \sum \nolimits_{GO \in {P_v}\left( t \right) - {T_v}} IC\left( {GO} \right).$$

## Materials

The following datasets and software versions were used:

- UniProt Knowledgebase release 2016-11 for DIAMOND searches
- Pfam-A release 30 for HMMER searches
- DIAMOND version 0.9.11.112
- HMMER version 3.3
- GOA UniProt data release 161 for creating the Wei2GO required data files
- GOA UniProt data release of 13-06-2020 for generating the test set
- go.obo release 02-12-2016
- pfam2go generated based on interpro2go release 31-10-2016
- Argot2 and Argot2.5 were last accessed on 08-04-2021
- DeepGOPlus was downloaded from https://github.com/bio-ontology-research-group/deepgoplus on 30-10-2020.

All datasets used by Wei2GO correspond to the datasets used by Argot2 and Argot2.5.

## Results

Wei2GO is made to facilitate open-source protein function prediction, and the ability to do this independent of web-servers. Two of these web-servers, Argot2.5 and its predecessor Argot2, utilize sequence similarity searches against the UniProtKB and Pfam databases, and apply a weighing algorithm to come to a final prediction score. Wei2GO takes that idea and puts this in an open-source and locally installable concept, which opens up the possibility to update data used by the algorithm independent of the original authors, and the ability to perform protein function prediction on big datasets.

### Algorithm comparisons between Wei2GO and Argot

Because Wei2GO’s concept is based on that of Argot, it is important to highlight major algorithmic differences between the different software (Table 1). Both Argot2 and Argot2.5 supplement pfam2go annotations with the GOA GO terms of all proteins belonging to each Pfam entry, which Wei2GO does not. Wei2GO weighs GO annotations differently based on the associated evidence codes in the GOA database, which both Argot’s do not. Argot2.5 is the only software that performs taxonomy-based filtering of GO terms. And finally, Argot2 and Argot2.5 can only handle 5,000 and 10,000 proteins at a time, respectively.

**Table 1:** An overview of the major algorithmic differences between Argot2, Argot2.5, and Wei2GO.

	Supplementing pfam2go annotations	Evidence-based weights	Taxonomy-based filtering	Limited number of input sequences
Argot2	Yes	No	No	Yes
Argot2.5	Yes	No	Yes	Yes
Wei2GO	No	Yes	No	No

To get an indication of the effect of the algorithmic differences between the software, Table 2 shows an overview of the correctly and incorrectly predicted protein-GO term pairs, irrespective of their prediction scores. The main difference between the software is the amount of false positive (FP) predictions. Argot2, which supplements pfam2go annotations, and to a lesser extend Argot2.5, which supplements pfam2go annotations and applies a taxonomic filter, show a substantially larger amount of FP predictions compared to Wei2GO whilst not showing a large increase in true positive (TP) predictions. Exceptions are Biological Processes (BPO) where Argot2.5 shows both a lower TP and FP count compared to Wei2GO, and Cellular Components (CCO) where Argot2 and Argot2.5 show an increase in TP count. This general trend suggests that supplementing Pfam annotations by adding GOA GO annotations from proteins belonging to the Pfam entry, which is not present in Wei2GO, adds quantity but not quality to the predictions.

**Table 2:** Comparison of Argot2, Argot2.5, and Wei2GO raw prediction counts. All predicted protein-GO term pairs present in the test set are classified as a True Positive (TP) and all predicted protein-GO term pairs not present in the test set are classified as False Positive (FP). Shown are the numbers for Biological Process ontology (BPO), Molecular Function ontology (MFO), and Cellular Component ontology (CCO).

	BPO TP	BPO FP	MFO TP	MFO FP	CCO TP	CCO FP
Argot2	39,781	1,766,545	3,325	122,286	19,325	238,918
Argot2.5	29,291	104,322	3,677	177,220	17,212	48,230
Wei2GO	39,460	119,702	3,467	33,815	10,903	33,748

### Performance comparisons between Wei2GO and Argot

Several performance metrics are used to compare Wei2GO and Argot: precision-recall curves, F_max_ scores, S_min_ scores, and computational time. DeepGOPlus is added as a part of all these analyses. This deep learning-based and open source protein function prediction software is an excellent example of both predictive performance and usability. Adding this software in the assessments provides perspective as to any performance differences between Wei2GO and Argot. For the purpose of this analysis, all databases used by Wei2GO are the same versions used by Argot2 and Argot2.5. Each species was given to Argot2.5 as a separate input with a species level taxonomic code.

**Figure 1:**
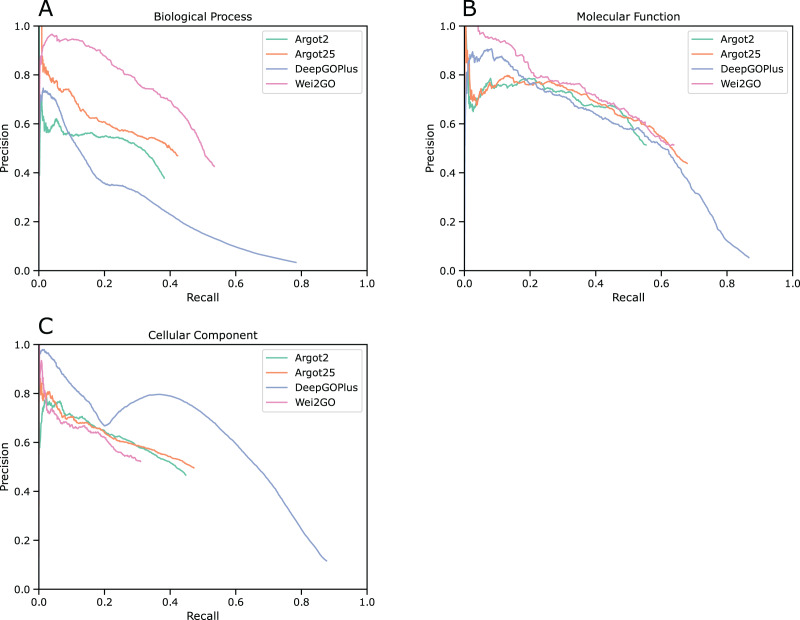
Precision recall curves for (A) biological process, (B) molecular function, and (C) cellular component ontologies.

Precision recall curves were generated for biological process (BPO), molecular function (MFO), and cellular component (CCO) ontologies (Fig. 1). This analysis shows that Wei2GO is the best performer in both the BPO and MFO assessment, with most notably BPO having a substantial increase in precision. For CCO, Wei2GO is slightly outperformed by Argot2, while DeepGOPlus performs substantially better than both.

F_max_ scores were calculated based on the highest F1-score at any given threshold, and S_min_ scores were calculated based on the lowest S-score at any given threshold (Table 3). The F_max_ score is highest in Wei2GO for BPO, similar between Wei2GO and Argot2.5 for MFO, and is outperformed by both Argot2 and DeepGOPlus for CCO. Wei2GO shows the lowest S_min_ score for both BPO and MFO, and is outperformed by both Argot2 and DeepGOPlus for CCO.

**Table 3:** Comparison of the maximum F1-scores (Fmax) and minimum S-scores (Smin) between methods for biological processes (BPO), molecular functions (MFO), and cellular components (CCO). Best results displayed in bold.

Method	Fmax BPO	Fmax MFO	Fmax CCO	Smin BPO	Smin MFO	Smin CCO
DeepGOPlus	0.32	0.56	**0.63**	90.31	11.71	22.71
Argot2	0.39	0.55	0.46	73.42	10.00	22.54
Argot2.5	0.45	**0.58**	0.48	69.01	9.72	**22.21**
Wei2GO	**0.52**	**0.58**	0.39	**53.08**	**8.87**	23.03

Wei2GO was compared to Argot2.5 on the CAFA3 data using the F_max_ score, relative to the top three performers of the challenge (Table 4). Both Wei2GO and Argot2.5 are competitive, and show similar results to those in Table 3. One notable difference is a discrepancy in performance for the BPO category. Protein function annotation is an ‘open world’ problem, *i.e*. a GO term annotation can be incorrectly assigned as a false positive due to an as-of-yet unidentified function of a protein (Dessimoz, Škunca & Thomas, 2013). The test set used for Table 3 is more complete for BPO terms than the CAFA3 challenge set, with 6.5 average leaf terms per protein compared to 3.6 respectively, resulting in a larger F_max_ difference between Wei2GO and Argot2.5. These differences in GO terms per protein were marginal for MFO and CCO terms.

**Table 4:** Comparison of the maximum F1-scores (Fmax) between the top three CAFA3 performers, Argot2.5, and Wei2GO. Given are the scores for biological processes (BPO), molecular functions (MFO), and cellular components (CCO). Argot2.5 scores are taken from the CAFA. Best results displayed in bold.

Method	Fmax BPO	Fmax MFO	Fmax CCO
CAFA #1	**0.40**	**0.62**	**0.61**
CAFA #2	0.39	0.54	**0.61**
CAFA #3	0.38	0.53	0.60
DeepGOPlus	0.39	0.56	**0.61**
Argot2.5	0.38	0.52	0.59
Wei2GO	0.39	0.52	0.57

Computational times were compared in Table 5 based on the 4,067 proteins of the test set. The Argot2 and Argot2.5 web-servers were run without queue times, and all analyses were run on one CPU.

**Table 5:** A comparison of the computational time required for the prediction of the test set used for the analysis in this paper. Based on 4,226 proteins. DIAMOND and HMMScan calculations required for the input of Argot2, Argot2.5, and Wei2GO, were excluded from the computational times. No queue times for Argot2 and Argot2.5 were reported.

Method	Computational time (min)
DeepGOPlus	2
Argot2	173
Argot2.5	303
Wei2GO	4

## Discussion

This paper describes Wei2GO: a weighted sequence similarity and python-based open source function prediction software. It utilizes a combination of DIAMOND and HMMER searches, and combines these to extract a probability score associated with GO terms. It is compared against the web-based tools Argot2 and Argot2.5, which share a similar concept, and DeepGOPlus, which acts as a reference point for the various metrics used.

Wei2GO shows a large improvement in predicting BPO terms according to the precision and recall, F_max_, and S_min_ evaluation metrics. Similarly, MFO shows a small improvement in the precision recall curve and S_min_ scores, but no difference in the F_max_ score compared to Argot2.5. CCO shows a slight decrease in performance over Argot2 and Argot2.5, and a substantial decrease in comparison to the reference DeepGOPlus.

In comparison to the reference, DeepGOPlus, Argot2 and Argot2.5 similarly show an improvement in BPO term prediction, which implies a sequence similarity-based approach is preferable over a deep learning-based approach. Since Argot and Wei2GO share a similar approach and used the same databases for their predictions, performance difference between them implies key algorithmic differences leading to a large improvement in BPO prediction for Wei2GO over Argot2 and Argot2.5. No definitive conclusion can be made due to the closed-source concept of Argot, however Table 2 shows Argot2 predicts many more GO terms than Wei2GO whilst not increasing the amount of correct predictions, which are then reduced by Argot2.5 due to presumed taxonomic filtering. This large increase is likely due to the Argot supplementing pfam2go annotations with the GOA annotations of all proteins sharing a Pfam entry.

MFO predictions are similar between Argot2, Argot2.5, Wei2GO, and the reference DeepGOPlus. While the same trend is shown in the number of correct and incorrect GO terms predicted by Argot2, Argot2.5, and Wei2GO as for BPO (Table 2), the precision and recall curve (Fig. 1), F_max_, and S_min_ scores (Table 3) show Argot2 and Argot2.5 are better at sorting out the incorrect predictions, leading to a much smaller increase in performance for Wei2GO.

Wei2GO performs worse than Argot2 and DeepGOPlus for CCO predictions. The big difference in precision between DeepGOPlus and the other methods indicates deep learning-based prediction is preferable for this ontology compared to sequence similarity-based prediction. A decrease in performance for Wei2GO compared to Argot2 and Argot2.5 could be due to the relative simple nature of the GO DAG for CCO compared to the other ontologies. Enhancing Pfam domains with the GOA GO terms of its protein members could in such a case improve the predictions. While the F_max_ difference between Argot2, Argot2.5, and Wei2GO is substantial, Fig. 1C indicates precision is similar and the decrease in performance is almost entirely due to a decrease in recall.

As observed in the precision recall curves (Fig. 1), Wei2GO offers a smoother curve for BPO and MFO predictions compared to Argot2 and Argot2.5. This is preferable, as it suggests better sorting of high-confidence and low-confidence predictions. While no definitive conclusion can be made due to the closed-source nature of Argot, this is likely a result of the evidence-based weighing of Wei2GO, as it is more reliable to transfer GO terms through sequence similarity when these come from experimental or curated evidence.

Finally, the computational time for Wei2GO is orders of magnitude lower than that of Argot2 and Argot2.5 (Table 5), while allowing more proteins to be predicted simultaneously. This opens up the possibility of high-throughput annotating large amounts of omics data with GO terms, as is often needed in the study of comparative genomics and big data studies.

Future implementations of Wei2GO will address two aspects. Firstly, to assess the impact of DIAMOND and HMMScan on Wei2GO, Fig. S1 shows a precision recall curve of Wei2GO using both DIAMOND and HMMScan, only DIAMOND, and only HMMScan input. Currently the HMMScan input provides recall but not precision to the Wei2GO predictions, indicating that threating HMMScan input different than DIAMOND input could be beneficial to final predictions, or that an alternative to HMMScan can provide improved results. Secondly, future versions of Wei2GO will allow for user-adjustable parameters, namely choosing between multiple semantic similarity algorithms and choosing between semantic similarity cutoffs used in the Wei2GO algorithm, as different ontologies or species could potentially benefit from a different parameter selection.

## Conclusion

Wei2GO offers an open-source, locally installable alternative of a proven concept for protein function prediction in the name of Argot2 and Argot2.5. Algorithmic differences in Wei2GO lead to a large performance increase of BPO predictions, while showing similar performance in MFO or CCO predictions. Computational time is decreased from hours down to minutes on a test set of 4,193 proteins, and contrary to Argot, Wei2GO is able to predict an unlimited amount of proteins at a time. In conclusion, Wei2GO not only provides a faster, more flexible, and open-source alternative to Argot2 and Argot2.5, but is able to substantially improve BPO predictions while producing similar performance for MFO and CCO.

### Implementation and availability

Wei2GO requires Python 3.6 or higher, and the NumPy package. Supplemental Files used to create the results in this paper, instructions on how to install the software, and instructions on how to run the software can be found on https://gitlab.com/mreijnders/Wei2GO. Additionally, Snakemake scripts are provided to run the full annotation pipeline, and update the databases to their latest versions.

## Supplemental Information

10.7717/peerj.12931/supp-1 Supplemental Information 1 Comparison of the maximum F1-scores (Fmax) and minimum S-scores (Smin) between Wei2GO only using DIAMOND input, only using HMMScan input, and using both as an input, for biological processes (BPO), molecular functions (MFO), and cellular components (CCO). Comparison of the maximum F1-scores (Fmax) and minimum S-scores (Smin) between Wei2GO only using DIAMOND input, only using HMMScan input, and using both as an input, for biological processes (BPO), molecular functions (MFO), and cellular components (CCO).

10.7717/peerj.12931/supp-2 Supplemental Information 2 Precision recall curves for (A) biological process, (B) molecular function, and (C) cellular component ontologies. Compared are Wei2GO with both DIAMOND and HMMScan as an input, Wei2GO with only DIAMOND as an input, and Wei2GO with only HMMScan as an input.

## Additional Information and Declarations

### Data Availability

The following information was supplied regarding data availability: The software and its Supplemental Files are available at GitLab: https://gitlab.com/mreijnders/wei2go.
