# Supplementary material for: Wei2GO: weighted sequence similarity-based protein function prediction
Source: PeerJ. 2022 Feb 15;10:e12931. doi: 10.7717/peerj.12931 (PMC8855713; doi:10.7717/peerj.12931)
Supplement: Supplemental Information 1 — Comparison of the maximum F1-scores (Fmax) and minimum S-scores (Smin) between Wei2GO only using DIAMOND input, only using HMMScan input, and using both as an input, for biological processes (BPO), molecular functions (MFO), and cellular components (CCO). [file peerj-10-12931-s001.docx]

Supplementary Table 1:

| Method | Fmax  BPO | Fmax  MFO | Fmax  CCO | Smin  BPO | Smin  MFO | Smin  CCO |
| --- | --- | --- | --- | --- | --- | --- |
| Wei2GO DIAMOND Only | **0.52** | 0.57 | 0.34 | **53.00** | **8.71** | **23.00** |
| Wei2GO HMMScan Only | 0.05 | 0.38 | 0.09 | 96.11 | 12.53 | 26.78 |
| Wei2GO | **0.52** | **0.58** | **0.39** | 53.08 | 8.87 | 23.03 |

Comparison of the maximum F1-scores (Fmax) and minimum S-scores (Smin) between Wei2GO only using DIAMOND input, only using HMMScan input, and using both as an input, for biological processes (BPO), molecular functions (MFO), and cellular components (CCO).
