# Supplementary figures and images for: Wei2GO: weighted sequence similarity-based protein function prediction
Source: PeerJ. 2022 Feb 15;10:e12931. doi: 10.7717/peerj.12931 (PMC8855713; doi:10.7717/peerj.12931)

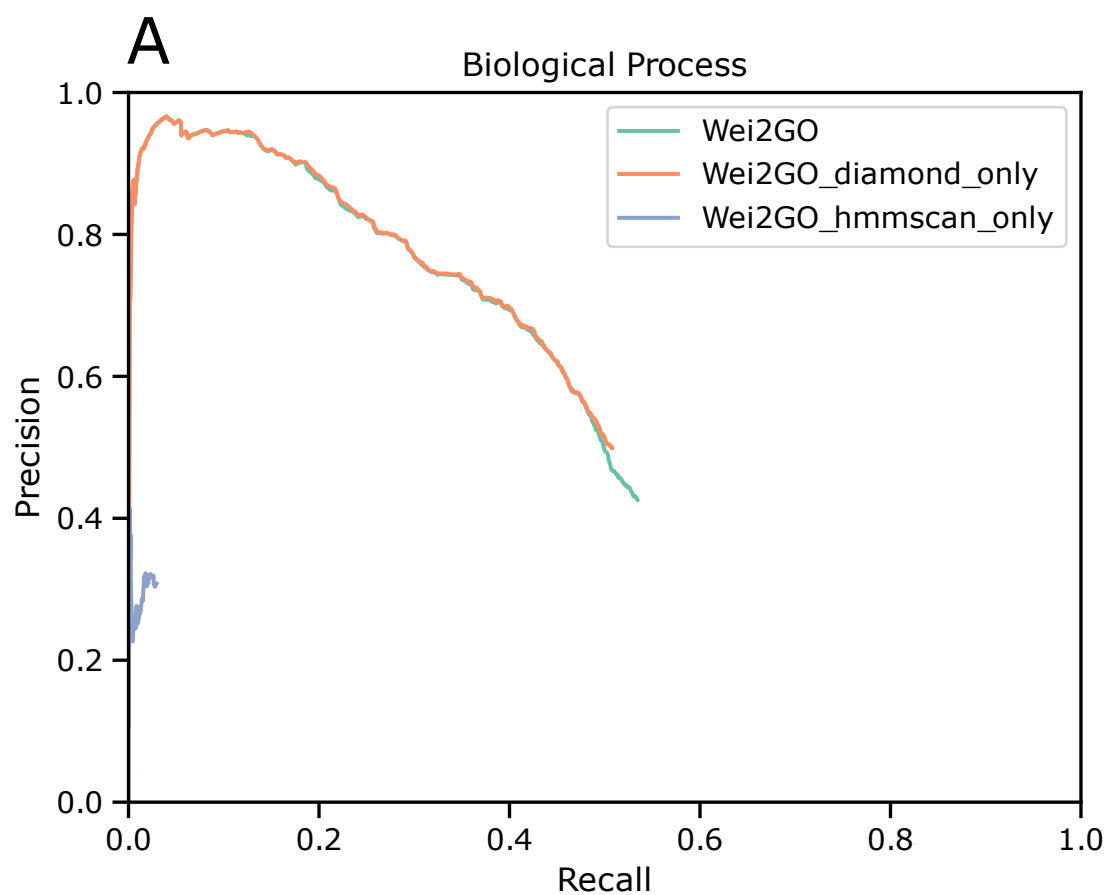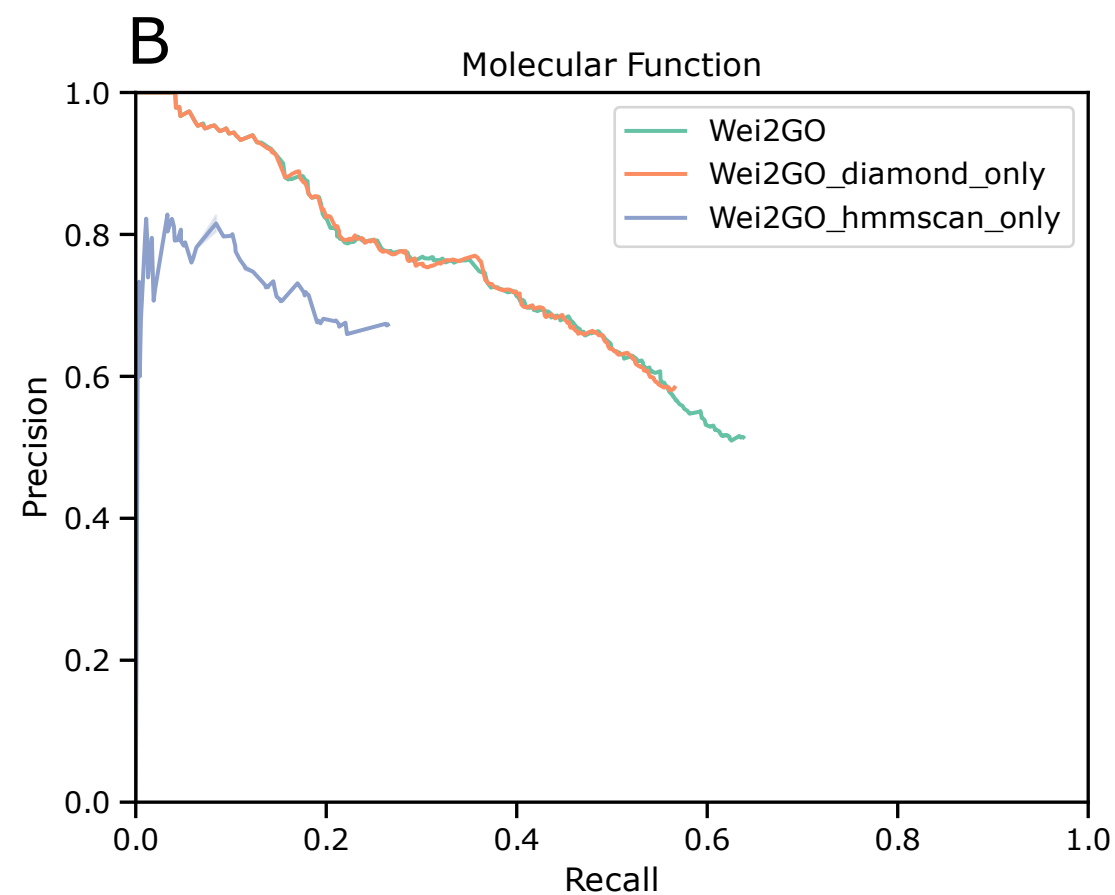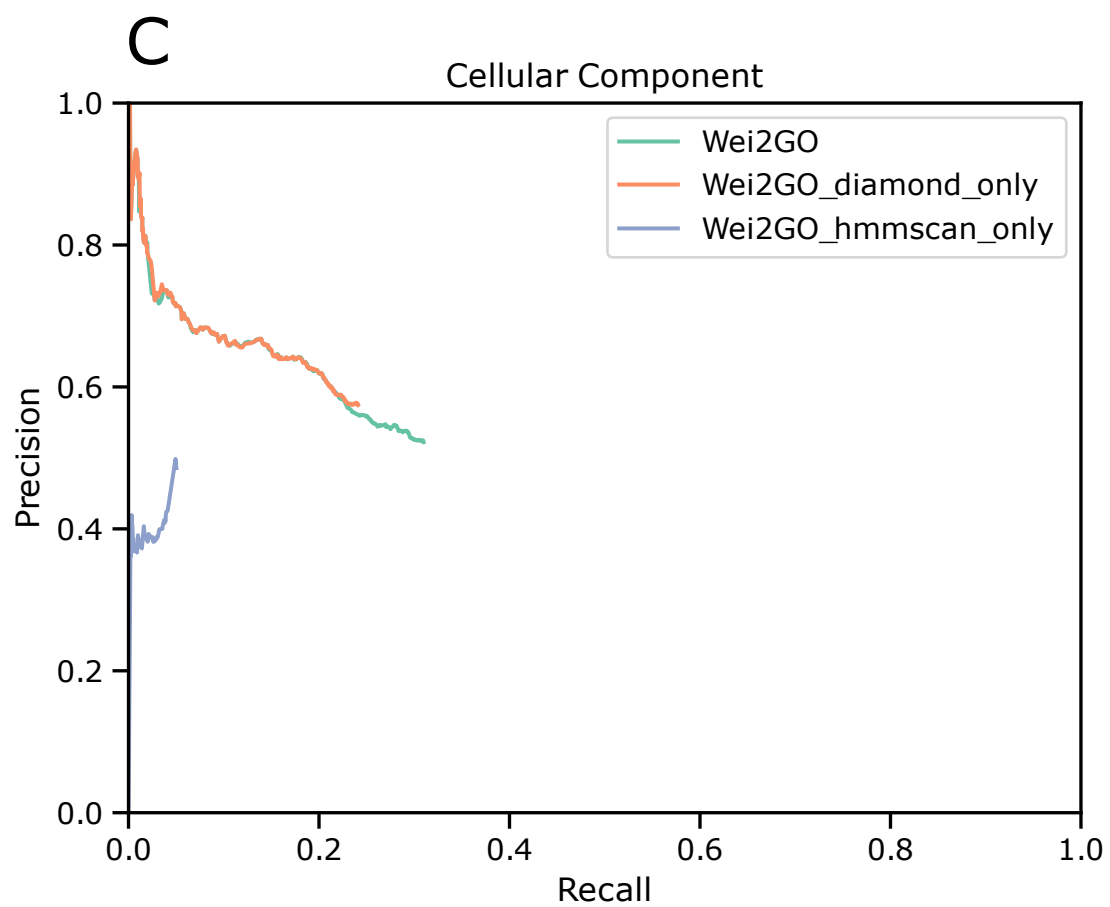

Supplement: Supplemental Information 2 — Compared are Wei2GO with both DIAMOND and HMMScan as an input, Wei2GO with only DIAMOND as an input, and Wei2GO with only HMMScan as an input. [file peerj-10-12931-s002.pdf]
